# Early versus late amniotomy during twin labor

**DOI:** 10.1002/ijgo.70803

**Published:** 2026-01-14

**Authors:** Or Eliner, Inbar Lidor, Or Touval, Gil Shechter Maor, Michal Kovo, Tal Biron‐Shental

**Affiliations:** ^1^ Department of Obstetrics and Gynecology Meir Medical Center Kfar Saba Israel; ^2^ Gray Faculty of Medical and Health Science Tel Aviv University Tel Aviv Israel; ^3^ Department of Obstetrics and Gynecology Hillel Yafe Medical Center Hadera Israel; ^4^ Department of Obstetrics and Gynecology Shamir Medical Center Be'er Ya'akov Israel

**Keywords:** amniotomy, cesarean delivery, neonatal morbidity, twin gestation

## Abstract

**Objective:**

This study evaluates whether timing of amniotomy affects labor characteristics and maternal and neonatal outcomes in twin deliveries.

**Methods:**

This retrospective study was conducted at a single academic medical center and included dichorionic diamniotic (DCDA) twin pregnancies with a normal anomaly scan and a vertex‐presenting leading twin, delivered over a 7‐year period. The cohort was divided into two groups: early amniotomy (performed at cervical dilation ≤3 cm) and late amniotomy (performed at >3 cm cervical dilation). Exclusion criteria included monochorionic twin pregnancies and planned elective cesarean deliveries. Maternal demographics, delivery characteristics, and neonatal outcomes were compared between groups.

**Results:**

Of 51 592 deliveries, 1196 were twins; 565 DCDA pregnancies met the inclusion criteria (early *n =* 279; late *n =* 286). Groups were similar in age, body mass index (kg/m^2^), comorbidities, and gestation. Nulliparity and intrapartum cesarean rates were more common in the early amniotomy group (56.6% vs. 29.4%; *P <* 0.001, 24.4% vs. 9.8%; *P <* 0.001, respectively). Induction and augmentation rates, rupture‐to‐delivery interval, second stage duration, intrapartum fever, and meconium were similar. Neonatal outcomes, including birthweight, 5‐min Apgar <7, neonatal intensive care unit admission, and composite morbidity were comparable. On adjusted analysis, early amniotomy (adjusted odds ratio [aOR] 1.84; 95% confidence interval 1.09–3.1), nulliparity (aOR 5.60; 3.17–9.87), and previous cesarean (aOR 3.94; 1.32–11.77) increased cesarean risk, whereas epidural was protective (aOR 0.40; 0.24–0.67).

**Conclusion:**

In DCDA twin labor, early amniotomy (≤3 cm) is associated with increased intrapartum cesarean, despite similar durations of labor and neonatal outcomes. Amniotomy timing should be individualized, with caution against routine early rupture.

## 1 INTRODUCTION

Twin pregnancies account for 1%–4% of all births, and their incidence has significantly increased over the past several decades.^1^, ^2^ The rise is largely driven by assisted reproductive technologies and delayed childbearing, both of which increase the likelihood of dizygotic twins.^1^, ^2^ Similar to singleton pregnancies, labor induction in twin pregnancies is often required before the spontaneous onset of labor due to maternal or fetal indications.^3^

Twin pregnancies carry a significantly higher risk of preterm birth (PTB) compared to singletons, affecting 40%–60% of twin pregnancies.^4^ While many PTBs in twin pregnancies are spontaneous, the rate of medically indicated PTBs has risen, largely due to the high incidence of complications such as hypertensive disorders of pregnancy and fetal growth restriction.^5^, ^6^ Further, perinatal mortality and morbidity rates in twin pregnancies are known to increase after approximately 38 weeks of gestation.^7^, ^8^ As a result, many clinical guidelines recommend elective delivery of twins between 36 and 39 weeks*'* gestation, depending on chorionicity and other perinatal factors.^9^, ^10^, ^11^

For patients with an unfavorable cervix, cervical ripening is often necessary. This can be achieved using mechanical methods (balloon catheter) or pharmacologic agents (prostaglandins); amniotomy (AROM) might initiate or augment labor but carries risks (cord prolapse, infection, fetal heart rate decelerations, and bleeding).^1^, ^12^, ^13^, ^14^, ^15^, ^16^, ^17^, ^18^

Evidence regarding the efficacy of amniotomy in singleton pregnancies remains mixed: reviews and trials show inconsistent effects on labor duration and no consistent effect on cesarean, leaving optimal timing debated.^12^, ^18^, ^19^, ^20^

Twin‐specific data on induction and intrapartum interventions are more limited than in singletons. Cohort studies and secondary analyses suggest that, beyond 36–37 weeks, induction in DCDA twins can be performed with acceptable maternal and neonatal outcomes when the first twin is cephalic and labor is managed in experienced units. Cesarean risk appears to depend more on parity and intrapartum conditions than on induction per se.^18^, ^21^, ^22^, ^23^, ^24^ Current guidance recommends planned delivery between 36 and 39 weeks by chorionicity, underscoring the routine need for active management in twins.^25^, ^26^, ^27^

In our institution, DCDA twin pregnancies are managed according to a standardized departmental protocol aligned with these recommendations. Planned vaginal birth is offered when Twin A is cephalic, both fetuses have reassuring status and estimated fetal weight 1500–4500 g, and no contraindications to vaginal twin birth are present (e.g., placenta previa or prior classical uterine incision). Labor is conducted with continuous electronic fetal monitoring of both fetuses, availability of regional anesthesia, and delivery in the operating room with anesthesia and neonatal teams present.

Given the unique physiological and clinical considerations in twin pregnancies, the success and safety profiles of labor induction methods might differ from those in singletons. Although substantial research exists on amniotomy in singleton pregnancies, data on its efficacy and safety in twin pregnancies are limited.^12^, ^13^, ^14^, ^16^, ^17^, ^25^, ^27^, ^28^, ^29^, ^30^, ^31^, ^32^ We therefore evaluated the efficacy and safety of early amniotomy during labor induction in dichorionic diamniotic (DCDA) twin labor. By examining maternal and neonatal outcomes, our article seeks to guide healthcare professionals in making evidence‐based decisions about the timing of amniotomy in twin labor. Ultimately, we aimed to contribute to the optimization of labor induction protocols, ensuring the best possible outcomes for mothers and their babies in twin pregnancies.

## 2 MATERIALS AND METHODS

### 2.1 Study design and setting

We conducted a retrospective cohort study of all dichorionic diamniotic (DCDA) twin pregnancies delivered at Meir Medical Center, a tertiary academic hospital in Kfar Saba, Israel, between January 2014 and December 2020. The institutional review board approved the protocol.

Institutional protocol for DCDA twin labor: Throughout 2014–2020, planned vaginal DCDA twin births at Meir Medical Center were managed according to a stable departmental protocol. Candidates for planned vaginal birth had a cephalic presenting Twin A and no contraindications to vaginal twin birth (e.g., placenta previa, prior classical uterine incision, or non‐vertex Twin A). In line with departmental criteria, a cephalic Twin A might be delivered vaginally across the usual twin weight spectrum (1500–4500 g). For Twin B, vaginal birth is permitted if cephalic; if non‐cephalic (breech or transverse), vaginal delivery is considered only when the estimated fetal weight is <4000 g and not more than 20% higher than the estimated weight of Twin A. Labor was conducted with continuous electronic fetal monitoring of both fetuses, regional anesthesia (epidural or spinal) available, and the active second stage and birth carried out in the operating room with anesthesia and neonatal teams present. The timing of amniotomy was left to the midwife*'*s or obstetrician*'*s clinical judgment; importantly, no changes to this protocol or to local twin‐labor guidelines occurred during the study period.

### 2.2 Participants

Inclusion criteria were gestational age 34–39 weeks, cephalic presentation of the first twin, both twins alive, and estimated fetal weight 1500–4000 g per fetus. Exclusion criteria were monochorionic twins, delivery <34 weeks, fetal chromosomal or structural anomalies, and contraindications to labor or vaginal birth (e.g., placenta previa, prior classical uterine incision, or more than one previous low‐segment cesarean). Women with one previous low‐segment transverse cesarean were eligible for a trial of labor in twin pregnancies, according to departmental protocol. In line with departmental criteria, a cephalic Twin A might be delivered vaginally across the usual twin weight spectrum (1500–4500 g). For Twin B, vaginal birth is permitted if cephalic; if non‐cephalic (breech or transverse), vaginal delivery is considered only when the estimated fetal weight is <4000 g and not more than 20% higher than the estimated weight of Twin A.

### 2.3 Exposure definition

Women entered the cohort with either spontaneous onset of labor or induction of labor. Spontaneous labor onset was defined as regular painful contractions with cervical dilation ≥2 cm occurring before any cervical‐ripening agent or oxytocin.

Induction of labor was defined as planned initiation of labor in the absence of regular painful contractions at admission, using cervical ripening (balloon catheter and/or prostaglandins) and/or oxytocin/AROM prior to the onset of regular contractions.

Augmentation of labor was defined as oxytocin infusion started after the onset of active labor (regular contractions with cervical dilation ≥2 cm) owing to inadequate uterine activity or slow progress, irrespective of whether labor began spontaneously or after induction.

Amniotomy timing was defined by cervical dilation at AROM for the first twin: early (≤3 cm) and late (>3 cm). Pregnancies with spontaneous rupture of membranes (ROM) and pre‐labor spontaneous rupture of membranes (PROM) were excluded.

#### 2.3.1 Determinants of amniotomy timing

The choice to perform AROM at ≤3 cm (early) versus >3 cm (late) was at the midwife/obstetrician's discretion, integrating parity, Bishop score (dilation/effacement/consistency), fetal head station/engagement, contraction adequacy, and the need for timely external/internal monitoring or induction sequencing. No fixed institutional cut‐off existed, and no departmental changes to AROM timing policy occurred during 2014–2020.

### 2.4 Data sources and variables

Maternal demographics, pregnancy characteristics, and neonatal outcomes were obtained from computerized medical records.

#### 2.4.1 Maternal data

Maternal data included age, gestational age at delivery, gravidity, parity, history of cesarean delivery (CD), in vitro fertilization (IVF) pregnancies, and body mass index (BMI, kg/m^2^).

Parity was recorded as the number of prior births ≥24 weeks. Women with one previous low‐transverse cesarean were eligible for trial of labor with DCDA twins if all other inclusion criteria were met; women with >1 previous cesarean or a prior classical uterine incision were excluded.

Parity was categorized as nulliparous versus multiparous, and previous cesarean delivery was recorded as a separate binary variable (0 or 1 previous low‐segment cesarean).

Gestational age was confirmed in all cases by first‐trimester ultrasonography. Maternal comorbidities included hypertensive disorders (chronic hypertension, pregnancy‐induced hypertension, and preeclampsia), defined according to the American College of Obstetricians and Gynecologists criteria,^33^ and diabetes mellitus, categorized as gestational diabetes (A1 and A2) or pregestational diabetes mellitus. Estimated fetal weight (EFW) was obtained from the last ultrasound examination before delivery. EFW in our work will be the mean EFW per fetus, calculated as the average of both twins*'* EFWs for each pregnancy.

#### 2.4.2 Delivery characteristics

Delivery characteristics included details of AROM for the first twin, cervical effacement and fetal head station at the time of AROM, color of amniotic fluid, duration of the second stage of labor, interval from ROM to delivery of the presenting twin (Twin A), and mode of delivery.

Induction of labor was defined as planned initiation using cervical ripening (balloon and/or prostaglandins) with or without oxytocin before the onset of regular contractions. Augmentation was defined as oxytocin started after labor onset for protraction or arrest.

Oxytocin augmentation was initiated according to departmental protocol when uterine contractions or cervical progress were judged inadequate after AROM.

#### 2.4.3 Neonatal characteristics

Neonatal characteristics and early neonatal outcomes were documented for each infant based on medical records and clinical assessments conducted immediately after birth by pediatricians. Neonatal outcomes included birth weight, 5‐min Apgar scores, umbilical cord blood pH, admission to the neonatal intensive care unit (NICU), hypoglycemia, sepsis, respiratory distress syndrome (RDS), need for phototherapy, anemia requiring blood transfusion, meconium aspiration syndrome, intraventricular hemorrhage (IVH), seizures, and hypothermia.

### 2.5 Outcomes

The primary outcome was intrapartum cesarean delivery of the presenting twin (Twin A), defined as any emergency cesarean performed after the onset of labor but before vaginal birth of Twin A, thereby resulting in cesarean delivery of both twins.

Indications for intrapartum cesarean delivery of the presenting twin (Twin A) were obtained from operative and labor records and classified as: arrest of dilation (no cervical change for ≥4 h in active labor despite adequate uterine contractions), arrest of descent of the presenting twin (no descent of the fetal head for ≥1 h in second stage with adequate contractions and maternal effort), non‐reassuring fetal heart rate (persistent category II–III tracing requiring expedited delivery according to departmental protocol), cord prolapse, suspected placental abruption, or other (e.g., combined or atypical indications).

Secondary outcomes included the total duration of labor (defined as the interval from admission to the delivery room to delivery), overall mode of delivery, intrapartum infectious morbidity (defined as chorioamnionitis or intrapartum fever), and, among women with vaginal birth of Twin A, urgent cesarean delivery for Twin B. Neonatal outcomes assessed included birth weight, 5‐min Apgar scores, presence of meconium‐stained amniotic fluid, neonatal sepsis, need for resuscitation, NICU admission, and a composite neonatal morbidity defined per infant as the occurrence of ≥1 of hypoglycemia, sepsis, RDS, transfusion, IVH, hypoxic–ischemic encephalopathy, or neonatal seizures.

### 2.6 Statistical analysis

Analyses were performed in SPSS v27.0 (IBM) and Python. Categorical variables were compared between groups using the Pearson *χ* ^2^‐test or Fisher*'*s exact test, as appropriate. Continuous variables were assessed for normality (Shapiro–Wilk) and variance equality (Levene) and compared with *t*‐tests or Mann–Whitney *U*, as appropriate.

A multivariable logistic regression model was constructed in which intrapartum cesarean delivery of the presenting twin served as the dependent variable, while nulliparity, induction of labor, augmentation, epidural, previous CD, and early amniotomy served as the independent variables. Potential confounders were selected based on prior research, clinical relevance, and their possible influence on both the risk of CD and neonatal outcomes. Results are reported as adjusted odds ratios (aOR) with 95% CIs; *P <* 0.05 was considered statistically significant.

The primary and secondary outcomes were compared between early and late amniotomy after cervical ripening under a prespecified analysis plan.

### 2.7 Ethics

This study was approved by the Meir Medical Center Institutional Review Board (protocol [ID] 0358‐20‐MMC); the requirement for informed consent was waived due to the retrospective design.

## 3 RESULTS

During the study period, a total of 51,592 deliveries occurred, of which 2.3% (1196) were twin pregnancies. Of these, 565 pregnancies (47%) met the eligibility criteria. Among the eligible cases, 279 pregnancies were categorized into the “early amniotomy” group and 286 into the “late amniotomy” group.

Table 1 compares maternal demographics and obstetric characteristics between the late and early amniotomy groups.

**TABLE 1:** Maternal demographic and obstetric characteristics of the studied groups.

	Early amniotomy group *N* = 279	Late amniotomy group *N* = 286	*P*‐value
Effacement	84.38 ± 15.96	84.44 ± 15.98	0.96
Head position	−1.86 ± 0.74	−1.85 ± 0.7	0.87
Maternal age (y)	31.9 ± 5.2	32.15 ± 4.6	0.54
Estimated fetal weight (g)	2378.5 ± 556.8	2409.7 ± 578.1	0.51
Nulliparity	158 (56.6)	84 (29.4)	**<0.001**
Previous CD	12 (4.3)	6 (2.1)	0.21
IVF pregnancy	40 (14.3)	33 (11.5)	0.76
BMI (kg/m^2^) >30	59 (21.1)	61 (21.3)	0.93
DM/GDM	52 (18.6)	50 (17.5)	0.8
Hypertensive disorders	23 (8.2)	25 (8.7)	0.9

*Note*: Continuous variables are presented as mean ± standard deviation and categorical variables as *n* (%). Effacement = cervical effacement (%) at the time of amniotomy. Head position = fetal head station in relation to the ischial spines (negative values indicate a station above the spines). Estimated fetal weight (EFW) = mean EFW per fetus (average of both twins) on admission ultrasound. Bold indicates statistically significant (*p* < 0.05).

Abbreviations: BMI, body mass index (kg/m^2^) at admission; CD, cesarean delivery; DM, diabetes mellitus, including GDM; GDM, gestational diabetes mellitus; IVF, in vitro fertilization.

Cervical effacement and fetal head station at the time of amniotomy were similar between groups.

Both groups had similar maternal age, gestational age, BMI >30, prior cesarean, rates of diabetes, hypertensive disorders, and IVF pregnancies. The estimated fetal weight did not significantly differ between the groups (2378.5 ± 556.8 g vs. 2409.7 ± 578.1 g, *P* = 0.51). The rate of nulliparity was significantly higher in the early amniotomy group (56.6% vs. 29.4%, *P* < 0.001).

Table 2 highlights differences in delivery characteristics between the late and early amniotomy groups.

**TABLE 2:** Delivery characteristics of the studied groups.

	Early amniotomy group *N =* 279	Late amniotomy group *N =* 286	*P*‐value
GA (weeks)	36.8 ± 1.2	37.0 ± 1.2	0.06
Induction	104 (37.3)	106 (37.1)	0.75
Augmentation	178 (63.8)	169 (59.1)	0.28
Epidural	237 (85)	252 (88.1)	0.33
Interval from ROM to delivery (h)	11.2 ± 1.5	11.1 ± 1.5	0.16
Meconium	8 (2.8)	16 (5.6)	0.14
Duration of the second stage of labor (min)	91 ± 5	90 ± 5	>0.99
Intrapartum fever	25 (8.9)	18 (6.3)	0.29
Intrapartum cesarean	68 (24.4)	28 (9.8)	**<0.001**
Urgent CD for Twin B (Twin A vaginally)	10 (3.58)	8 (2.79)	0.6

*Note*: Continuous variables are presented as mean ± standard deviation and categorical variables as *n* (%). Intrapartum fever >38°C. Intrapartum cesarean = cesarean delivery of leading baby. Urgent CD for Twin B is calculated among cases with a vaginal birth of Twin A. Bold indicates statistically significant (*p* < 0.05).

Abbreviations: CD, cesarean delivery; GA, gestational age; ROM, rupture of membranes.

The rates of labor induction and augmentation were comparable between the groups.

Intrapartum cesarean delivery was significantly more frequent in the early amniotomy group (24.4% vs. 9.8%, *P <* 0.001). Urgent cesarean delivery for Twin B after vaginal birth of Twin A was uncommon and did not differ significantly between groups (2.8% vs. 3.6%, *P* = 0.60).

Among women who underwent intrapartum cesarean delivery, indications differed by amniotomy timing (Table 3). Arrest of descent of the presenting twin was significantly more frequent in the late amniotomy group compared with the early group (21.4% vs. 2.9%, *P* = 0.003), whereas arrest of cervical dilation was more common in the early group (22.3% vs. 14.3%, *P* = 0.31). Non‐reassuring fetal heart rate was the most frequent indication overall, with no significant difference between groups (46.4% vs. 36.8%, *P* = 0.38). Other indications, including cord prolapse, suspected abruption, and miscellaneous causes, were rare and similar between groups.

**TABLE 3:** Indications for intrapartum cesarean delivery of the presenting twin (Twin A) by timing of amniotomy.

Reason for intrapartum cesarean	Early amniotomy group *N =* 68	Late amniotomy group *N* = 28	*P*‐value
Arrest of descent	2 (2.9)	6 (21.4)	**0.003**
Arrest of dilation	16 (23.5)	4 (14.3)	0.31
Cord prolapse	1 (1.5)	1 (3.6)	1.0
Non‐reassuring fetal heart rate	25 (36.8)	13 (46.4)	0.38
Suspected abruption	1 (1.5)	0 (0)	1.0
Other indication	3 (4.4)	0 (0)	0.26

*Note*: Categorical variables presented as *n* (%). Intrapartum cesarean = cesarean delivery of leading baby. Bold indicates statistically significant (*p* < 0.05).

The interval from ROM to delivery of Twin A and the duration of second stage were similar (11.2 ± 1.5 h vs. 11.1 ± 1.5 h; *P* = 0.16; 91 ± 5 min vs. 90 ± 5 min; *P >* 0.99). Intrapartum fever and presence of meconium did not differ.

Table 4 shows neonatal outcomes per infant. There were no significant differences in neonatal outcomes between the late and early amniotomy groups: birth weight was similar; Apgar <7 and NICU admission were rare and comparable; cord pH <7 showed a non‐significant trend with early amniotomy (0.8% vs. 0; *P* = 0.06); composite morbidity was 3.7% vs. 3.8% (*P* = 0.80).

**TABLE 4:** Comparison of neonatal outcome between the groups.

	Early amniotomy group *N =* 558	Late amniotomy group *N* = 572	*P*‐value
Birth weight (g)	2546.8 ± 402	2547.3 ± 401	>0.99
Apgar 5 min <7	1 (0.2)	2 (0.3)	>0.99
Cord pH <7	4 (0.8)	0	0.06
Admission to NICU	21 (3.6)	14 (2.7)	0.3
Hypoglycemia	2 (0.3)	5 (0.8)	0.4
Sepsis	1 (0.2)	0	0.5
Respiratory distress	0	0	>0.99
Phototherapy	16 (2.8)	16 (2.8)	>0.99
Blood transfusion	1 (0.2)	1 (0.1)	>0.99
Hypothermia	0	0	>0.99
Composite adverse neonatal outcome	19 (3.7)	22 (3.8)	0.8

*Note*: *N* = per infant. Continuous variables are presented as mean ± standard deviation and categorical variables as *n* (%). Hypoglycemia <36 mg/dL (<2.0 mmol/L). Composite adverse neonatal outcome was defined per infant as the presence of at least one of the following: hypoglycemia, sepsis, respiratory distress, anemia/blood transfusion, intraventricular hemorrhage, hypoxic–ischemic encephalopathy, or convulsions.

Abbreviation: NICU, neonatal intensive care unit.

Table *5* *outlines* multivariable logistic regression analysis for intrapartum cesarean delivery. Early amniotomy (aOR 1.84, 95% confidence interval [CI] 1.09–3.10; *P* = 0.02), nulliparity (aOR 5.60, 95% CI 3.17–9.87; *P <* 0.001), and previous cesarean (aOR 3.94, 95% CI 1.32–11.77; *P* = 0.01) were independent predictors, whereas epidural was associated with reduced odds (aOR 0.40, 95% CI 0.24–0.67; *P <* 0.001). Induction and augmentation were not independently associated with intrapartum cesarean.

**TABLE 5:** Multivariable logistic regression analysis for intrapartum cesarean delivery.

Variable	Adjusted odds ratio	95% CI for OR		*P*‐value
		Lower	Upper	
Nulliparity	5.6	3.17	9.87	**<0.001**
Induction of labor	1.28	0.75	2.19	0.35
Augmentation	0.73	0.42	1.29	0.28
Epidural	0.4	0.24	0.67	**<0.001**
Previous CD	3.94	1.32	11.77	**0.01**
Early amniotomy	1.84	1.09	3.1	**0.02**

*Note*: Bold indicates statistically significant (*p* < 0.05).

Abbreviations: aOR, adjusted odds ratio; CD, cesarean delivery; CI, confidence interval.

## 4 DISCUSSION

This study comparing early versus late amniotomy in DCDA twin pregnancies provides new evidence on intrapartum management, with implications for mode of delivery and neonatal safety.

### 4.1 Main findings

Baseline maternal and obstetric characteristics were largely comparable between groups. Early amniotomy was more frequently performed in nulliparous women, consistent with clinical practice patterns reported previously.^2^, ^23^, ^34^ Despite similar rates of induction and augmentation and no differences in the interval from rupture of membranes to delivery or second‐stage duration, intrapartum cesarean was significantly more common after early amniotomy. In adjusted analyses, early amniotomy, nulliparity, and previous cesarean independently increased cesarean odds, whereas epidural analgesia was associated with lower odds.

Among intrapartum cesareans, arrest of descent of the presenting twin was more frequent after late amniotomy, whereas arrest of cervical dilation was more frequent after early amniotomy. Neonatal outcomes, including composite morbidity, did not differ between groups.

### 4.2 Interpretation

#### 4.2.1 Maternal and obstetric characteristics

Groups were well matched on key baseline risks (BMI, hypertensive disorders, and gestational age), supporting internal validity. Nulliparity was more common with early amniotomy, a factor independently associated with intrapartum cesarean delivery in our model (Table 4). The higher proportion of nulliparas in the early AROM group aligns with reports that clinicians intervene earlier in first births to mitigate dystocia risk in twins.^2^, ^23^, ^34^ In our study, nulliparity was independently associated with intrapartum cesarean (Table 4).

For instance, Chasen and Chervenak (2017) highlighted the need for individualized labor management in twins,^35^ emphasizing that amniotomy timing should consider maternal parity and labor dynamics.

These insights resonate with our findings and underscore the importance of tailored clinical decisions.

#### 4.2.2 Delivery characteristics

The observation of higher intrapartum cesarean delivery rates in the early amniotomy group (*P* < 0.001) raises important considerations regarding the risks associated with early intervention. This observation suggest that early amniotomy, while intended to facilitate labor progression, might inadvertently increase the likelihood of non‐reassuring fetal status or labor complications requiring surgical intervention. Prior studies have demonstrated this association.^13^, ^16^, ^28^

The similarity in effacement, fetal head station at the time of amniotomy, second‐stage duration, and meconium‐stained fluid between groups suggests that the higher intrapartum cesarean rate with early amniotomy is not simply a surrogate for more advanced or unfavorable labor conditions at baseline but might reflect the impact of timing itself in DCDA twin labor.

While early amniotomy was independently associated with a higher overall cesarean rate, late amniotomy appeared more prone to mechanical arrest at the descent phase. The findings suggest that the timing of membrane rupture influences the mechanics of twin labor rather than simply accelerating it. With intact membranes, the hydrostatic wedge might persist longer, delaying engagement and descent (consistent with the greater proportion of arrest of descent after later amniotomy), whereas early rupture reduces the cushioning effect of the sac, potentially hastening descent but unmasking ineffective uterine activity, reflected by the higher proportion of arrest of dilation.

This concept is supported by singleton data describing the role of the membranes in facilitating descent.^20^ In twins, however, uterine overdistension, inter‐twin dynamics, and variable second‐twin presentation likely blunt the theoretical efficiency gains of early rupture and might increase the propensity to operative delivery.^29^ Unlike singleton pregnancies, twins inherently involve greater mechanical complexity and labor unpredictability, which might counteract the theoretical advantages of early membrane rupture.

Observational twin cohorts have linked earlier intrapartum interventions with higher cesarean rates,^29^ whereas randomized trials and meta‐analyses in singletons report mixed effects on time to delivery and generally no increase in cesarean with earlier amniotomy, sometimes with shorter labors.^12^ Our findings therefore caution against extrapolating singleton benefits to twins.

Importantly, the rate of mixed delivery (vaginal Twin A followed by urgent cesarean for Twin B) was low and similar between early and late amniotomy, suggesting that amniotomy timing did not materially affect the risk of cesarean delivery for the second twin in our cohort; however, the small number of such events limits the power of this secondary analysis.

#### 4.2.3 Labor efficiency

Other labor metrics (augmentation, induction, second stage, and rupture‐to‐delivery interval) were similar by timing, indicating that earlier rupture did not meaningfully shorten labor in this twin population. This aligns with the broader message from the Twins Timing of Birth Trial that intrapartum interventions must balance efficiency against the risk of emergent cesarean^24^ and with studies showing that early rupture does not necessarily increase subsequent intervention needs.^36^

Our multivariable findings reinforce established risk factors for intrapartum cesarean in twins: nulliparity and prior cesarean were strong predictors.^22^, ^37^ The observed protective association of epidural against intrapartum cesarean contrasts with historical concerns about dystocia^21^, ^38^ and might indicate that effective analgesia facilitates tolerance of longer labor and optimizes maternal effort with vigilant monitoring in twin units.

Our findings differ from much of the literature on amniotomy in singleton pregnancies. Randomized trials and meta‐analyses evaluating early versus late amniotomy after cervical ripening in singleton inductions have generally shown shorter induction‐to‐delivery intervals without a consistent increase in cesarean delivery, and some have reported modest reductions in time to birth when amniotomy is performed early in active labor.^15^, ^19^, ^28^, ^30^, ^31^ In contrast, in this DCDA twin cohort, early amniotomy (≤3 cm) was independently associated with a higher intrapartum cesarean rate despite similar labor duration and comparable neonatal outcomes. This divergence likely reflects the distinct biomechanics and clinical contingencies of twin labor (uterine overdistension, inter‐twin interactions, and the need to maintain a safe scenario for delivery of a second fetus), which might negate the theoretical efficiency gains of early membrane rupture observed in singletons.

Twin‐specific data on induction and intrapartum interventions are more limited, but observational studies of induced DCDA twins suggest that cesarean risk is largely driven by parity, presentation, and intrapartum complications rather than the induction method itself.^23^, ^24^, ^38^, ^39^ Our results are consistent with this broader picture: after adjustment for parity and previous cesarean, early amniotomy still conferred excess intrapartum cesarean risk, while neonatal morbidity remained low and similar between groups. Taken together, these data suggest that early amniotomy might shift the risk–benefit balance unfavorably in twins, supporting a more conservative approach to membrane rupture timing in this population.

#### 4.2.4 Neonatal outcomes

Neonatal outcomes were comparable across groups. Birthweight, 5‐min Apgar <7, NICU admission, and composite morbidity did not differ, and the higher frequency of cord pH <7 in the early group did not reach significance. These findings, in the context of active intrapartum surveillance, suggest that amniotomy timing does not materially affect short‐term neonatal risk in DCDA twins. This is consistent with studies indicating that neonatal outcomes in twins are driven more by gestational age, presentation, and intrapartum management than by amniotomy timing per se.^13^, ^17^, ^30^ The non‐significant trend toward lower cord pH with early rupture mirrors patterns reported elsewhere^40^ and might justify heightened fetal surveillance when early amniotomy is chosen, without implying harm when appropriate monitoring is in place. Earlier reports linking early rupture to infectious or hypoxic morbidity^13^, ^26^, ^41^ were not corroborated here.

Importantly, the lack of significant differences in adverse neonatal outcomes reinforces the safety of both approaches (early/late AROM) under skilled obstetric management.

### 4.3 Clinical implications and recommendations

For DCDA twin labor, routine early amniotomy (≤3 cm) should be used cautiously: it is associated with higher intrapartum cesarean without clear neonatal advantage. A more conservative (later) approach is reasonable when maternal–fetal status allows, particularly in multiparous patients or those with favorable cervixes and reassuring labor progress. Where early rupture is preferred (e.g., to facilitate monitoring access or within specific induction protocols), close surveillance is prudent.

These data support a cautious, individualized approach to the timing of amniotomy, rather than routine early rupture in all cases. Clinicians should weigh the potential benefits of early ROM, such as expedited labor progression, against the increased likelihood of surgical intervention.

Future research should focus on randomized controlled trials to confirm these findings and focus on refining criteria for amniotomy timing, particularly in subgroups with differing baseline risks.

### 4.4 Strengths and limitations

Strengths include a large, well‐defined DCDA cohort, clear exposure definition, consistent single‐center intrapartum protocols, and prespecified covariate adjustment. Labor management was consistent among participants because they were all delivered in a single academic institution and were managed according to departmental protocol.

Limitations inherent to retrospective design include residual confounding and potential selection bias in the choice and timing of amniotomy. We did not evaluate longer‐term neonatal outcomes, and results might not generalize to monochorionic twins or settings with different intrapartum protocols. Prospective, ideally randomized, comparisons of amniotomy timing in twins are needed, with stratification by parity, cervical status, and first‐twin station and with rigorous neonatal follow‐up.

The primary outcome captured intrapartum cesarean of the presenting twin, resulting in cesarean birth of both twins. The number of urgent cesarean deliveries for Twin B after vaginal birth of Twin A was small, limiting our ability to draw firm conclusions about second twin‐specific risks by amniotomy timing.

We did not perform a dedicated VBAC subgroup analysis due to the small number of women with a prior cesarean (18/565, 3.2%; six in the late amniotomy group and 12 in the early group), which limits conclusions regarding amniotomy timing specifically in twin TOLAC.

In addition, the interval between AROM and initiation of oxytocin was not reliably captured and could not be examined, which might have influenced labor progression; however, we believe it is unlikely to fully explain the robust association between early AROM and intrapartum cesarean.

## 5 CONCLUSION

In DCDA twin labor, early amniotomy (≤3 cm) was associated with a higher intrapartum cesarean rate, with no detectable neonatal disadvantage compared with later amniotomy, and did not shorten labor in this retrospective cohort.

Given the similar labor metrics and neonatal outcomes, routine early rupture might offer limited benefit and could increase the likelihood of surgical delivery.

Clinicians should individualize the timing of amniotomy, favoring a more conservative (later) approach when maternal and fetal conditions allow and applying vigilant monitoring whenever early rupture is deemed necessary. Further prospective studies should refine selection criteria and evaluate longer‐term infant outcomes to optimize intrapartum strategies in twin gestations.

## AUTHOR CONTRIBUTIONS

O.E. and T.B.‐S. conceived the study and supervised analysis. O.E. and M.K. designed the study. O.T. and I.L. collected data. O.E. and O.T. performed/oversaw statistical analyses. T.B.‐S. and G.S.M. provided clinical oversight. All authors interpreted results, revised the manuscript, and approved the final version.

## CONFLICT OF INTEREST STATEMENT

The authors declare that they have no competing interests.

## FUNDING STATEMENT

The authors have nothing to report.

## ETHICS APPROVAL AND CONSENT TO PARTICIPATE

This study was performed in line with the principles of the Declaration of Helsinki. All methods were carried out in accordance with relevant guidelines and regulations. All experimental protocols were approved by Meir Medical Center Human Investigation/Ethics Committee number 0358‐20‐MMC. Due to the retrospective nature of the study, Meir Medical Center Human Investigation/Ethics Committee has waived need for informed consent for the study.

## Data Availability

The data underlying this article are available from the corresponding author upon reasonable request and with permission from Meir Medical Center and Clalit Institute.
